# Hyaluronidase and pH Dual-Responsive Nanoparticles for Targeted Breast Cancer Stem Cells

**DOI:** 10.3389/fonc.2021.760423

**Published:** 2021-12-24

**Authors:** Weinan Li, Xiaoyu Zhang, Yang Nan, Li Jia, Jialin Sun, Lina Zhang, Yanhong Wang

**Affiliations:** ^1^ School of Pharmacy, Heilongjiang University of Chinese Medicine, Harbin, China; ^2^ Department of Pharmacy, Heze Medical College, Heze, China; ^3^ Biological Science and Technology Department, Heilongjiang Vocational College for Nationalities, Harbin, China

**Keywords:** hyaluronic acid, pH-responsive, hyaluronidase-responsive, cancer stem cells, CD44 receptors

## Abstract

pH-responsive and CD44 receptor-mediated targeted nanoparticles for eliminating cancer stem cells (CSCs) were developed based on complexes of PEG-poly(β-amino ester) (PEG-PBAE) micelles (PPM) coated with hyaluronic acid (HA) (HA-coated PPM complex, or HPPMc). Thioridazine (Thz) was loaded into HPPMc with a decent drug loading content. The release results of the drug *in vitro* showed that Thz was released from the HPPMc, which was stimulated by both the acidic pH and specific enzymes. Cytotoxicity studies on mammospheres (MS) revealed that the toxicity potential of Thz-loaded HPPMc (Thz–HPPMc) at pH 5.5 was better than drug solutions. Compared with that at pH 7.4, a higher cellular uptake of a coumarin-6 (C6)-labeled complex at pH 5.5 was observed, which demonstrated that complexes were efficiently taken up in MS. Meanwhile, free HA competitively inhibited the cellular uptake of HPPMc, which revealed that the uptake mechanism was CD44 receptor-mediated endocytosis. Within the acidic endolysosomal environment, the protonation of PBAE facilitated the escape of the complex from the lysosome and releases the drug. The results of *in vivo* distribution studies and tumor suppression experiments showed that HPMMc could stay in the tumor site of BALB/c nude mice for a longer period of time, and Thz–HPPMc could significantly improve the tumor-suppressing effect. All these results demonstrated the great potential of the multifunctional nanoparticle system for eliminating CSCs.

**Graphical Abstract:**
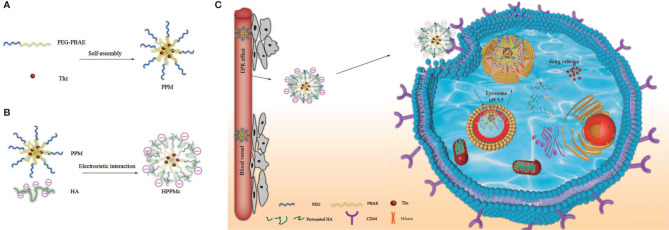


## 1 Introduction

Recently, the cancer stem cells (CSCs) theory has received sustained and increased attention because it explains relapse and metastasis in a range of carcinomas, including breast cancer stem cells (BCSCs) (1). Despite advances in diagnosis and treatment, breast cancer still has a very high mortality rate (2). With the development of the CSCs theory, it has been well known that traditional chemotherapy and radiotherapy can kill differentiated cancer cells rather than undifferentiated CSCs (3). Therefore, how to kill CSCs that cause cancer recurrence and metastasis has become a new targeted strategy for antitumor therapy.

BCSCs are marked as a CD44^+^/CD24^−^ phenotype, which means that CD44 is expressed in BCSCs, but CD24 is not (4). CD44 is a non-kinase transmembrane glycoprotein. It is related to the proliferation, differentiation, and migration of cancer cells (5, 6). In terms of breast cancer, when CD44 was knocked down in a CD44^+^/CD24^−^ breast cancer cell subpopulation, BCSCs differentiated into non-BCSCs with low tumor potential and altered the cell cycle and expression profiles of some stem cell-related genes (7). Abraham et al. have confirmed that CD44 expression was upregulated in breast cancer with bone or lung metastasis (8). Brown et al. also reported that CD44 levels were elevated in high-grade human breast tumors (9). Previous studies have shown that targeting the CD44 receptor of BCSCs is able to reduce the recurrence of breast cancer (10, 11). Thus, CD44 plays a very important role in drug targeting therapy of BCSCs.

Hyaluronic acid (HA), a natural polysaccharide, is biodegradable, biocompatible, non-immunogenic, and non-toxic (12). Moreover, HA specifically binds to CD44 receptors overexpressed on the surface of breast tumor cells and BCSCs (13). Hyaluronidase (HAase), an endogenous glycosidase, plays a leading role in tumor development, invasion, and metastasis (14). In addition, HA can also be rapidly degraded by HAase-rich endosomes (15).

Thioridazine (Thz) is a piperidine antipsychotic drug that can inhibit CSCs in a variety of cancers, such as gastric cancer, cervical cancer, and liver cancer (16–18). Recent studies have shown that Thz can interfere with the function of STAT3 and inhibit the activity of BCSCs, but has no effect on normal stem cells (19, 20). However, Thz is well tolerated at a low dose, but a Thz overdose leads to adverse reactions such as involuntary exercise and severe dizziness. In addition, free Thz has a number of problems such as poor solubility, nonspecific cardiotoxicity, and even pigmented retinopathy (21). Targeted delivery of Thz loaded into nanoparticles is able to overcome these challenges (22).

In tumor-targeting therapy, it plays a critical role in the rapid intracellular release of the drug loaded into the nanoparticles. In current research on tumor-targeted strategies, drug delivery carriers constructed by physical stimulation (such as pH and temperature) or enzymatic reactions have obvious special potential. But the combined application of pH and enzyme reaction to design drug delivery systems is not common in BCSCs-targeted research. Previously, we prepared pH-sensitive PEG-poly(β-amino ester) (PEG-PBAE) micelles (PPM) for targeting BCSCs (23). All of the results showed that, on account of the protonation of PBAE, the micelles presented certain cytotoxicity, efficient internalization, and rapid release of the drug triggered by pH in BCSCs, which were able to achieve an effective accumulation of intracellular drug concentration.

**Scheme 1:**
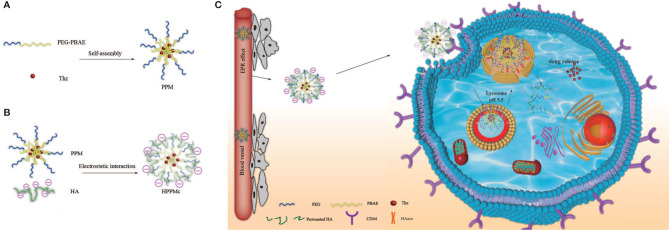
Schematic diagram of hyalurunic acid (HA)-coated self-assembly PEG-poly(β-amino ester) (PEG-PBAE) micelles and the process of intracellular drug delivery under acidic and hyaluronidase (HAase) conditions. **(A)** Thioridazine (Thz)-loaded micelles prepared in an aqueous environment based on PEG-PBAE. **(B)** The complex of HA-coated PEG-PBAE micelles prepared by electrostatic interaction. **(C)** Due to CD44 receptor-mediated endocytosis, the complex is selectively taken up by cancer stem cells and delivered to lysosomes, and Thz is transported into the cytoplasm in the presence of acidity and HAase, which improves the release of intracellular drugs and the therapeutic effect on cancer stem cells (CSCs).

In this study, we aimed to construct a novel drug targeting CSCs and a dual-responsive drug delivery system, which is a complex of HA modified the Thz–PPM programmed drug release behavior to specifically recognize and selectively kill BCSCs overexpressing CD44 under stimulation of pH and HAase (**Scheme 1**). The goal was to achieve an endosomal endoenzyme-responsive and a pH-responsive release in the lysosome after endocytosing the complex through the CD44 receptor.

## 2 Experimental

### 2.1 Materials

HA (*M*_n_ = 9,895) was purchased from Shangdong Huaxi Furuida Biomedical Co., Ltd. (Jinan, China). The ingredients for PPM including 1,6-hexanediol diacrylate (HDD), 4-methylpiperidine (MP), 1,3-bis(4-piperidyl)propane (TDP), and Thz were obtained from Alfa Aesar Chemistry Co., Ltd. (Beijing, China). Shanghai Ponsure Biotechnology Co., Ltd. (Shanghai, China) provided polyethylene glycol bis(amine) (NH_2_-PEG-NH_2_, *M*_n_ = 2,000). Coumarin-6 (C6) can be purchased from Shanghai Aladdin Biochemical Technology Co., Ltd. (Shanghai, China). 3-(4,5-Dimethylthiazol-2-yl)-2,5-diphenyl tetrazolium bromide (MTT) was from Sigma (St. Louis, MO, USA). Some cell culture media and reagents, including Dulbecco’s modied Eagle’s medium (DMEM), fetal bovine serum (FBS), dye dimethylindole red (Dir), and trypsin, were obtained from Beyotime Biotechnology (Shanghai, China). Fibroblast growth factor (FGF) and human epidermal growth factor (human EGF) were obtained from Pepro Tech Inc. (Rocky Hill, NJ, USA). The Lyso tracker and Hoechst 33258 were purchased from Beyotime Biotechnology. All solvents and reagents were of analytical grade and used without further purification.

Shanghai Fuheng Biological Co., Ltd. (Shanghai, China) provided human breast cancer cells (MCF-7). To obtain mammospheres (MS), MCF-7 cells in the logarithmic growth phase were grown in DMEM-F12 serum-free medium containing human EGF and FGF at 37°C for 20 days. The final MS were collected at 1,000 rpm for 5 min (24, 25).

BALB/c nude mice were purchased from the Medicine Laboratory Animal Center of Heilongjiang University of Chinese (Harbin, China). All animal experimental protocols were conducted in accordance with the guidelines specified for “biological and medical experimentation” at Heilongjiang University of Chinese Medicine (item identification code: HUCM-LS2019-06-15-101; date of approval: June 15, 2019).

### 2.2 Preparation of Thz–HPPMc

#### 2.2.1 Loading of Thz Into PPM

**Scheme 2:**
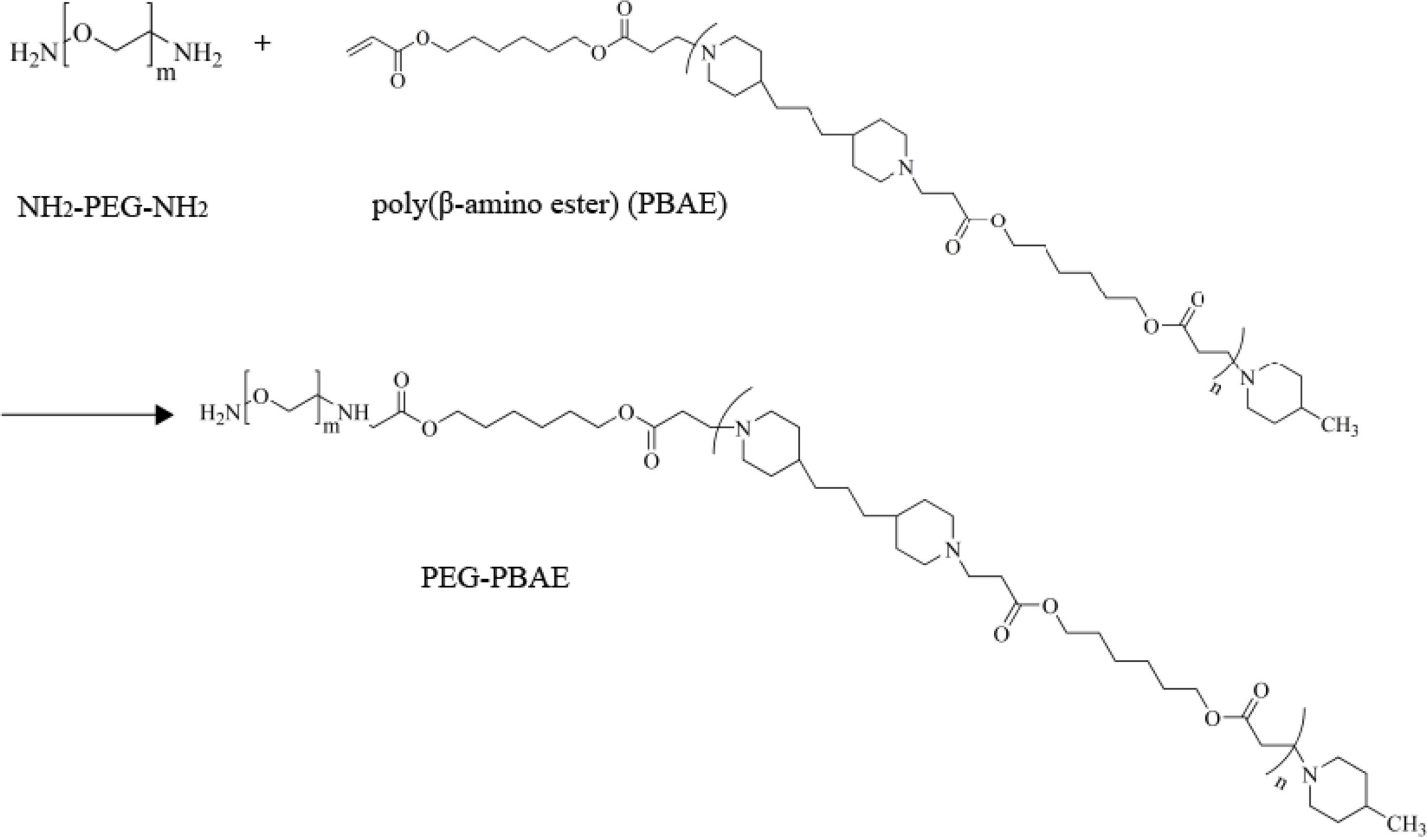
Synthesis route of the PEG-poly(β-amino ester) (PEG-PBAE) micelle (PPM).

In **Scheme 2**, PPM was synthesized *via* a Michael-type step polymerization, which we previously reported (23). Briefly, PBAE and PEG (Eq. 1) were dissolved in chloroform and stirred in an oil bath at 60°C for 48 h. After the reaction, the solution was concentrated and transferred to a dialysis bag (*M*_w_ = 5,000), dialyzed in distilled water for 48 h, and freeze dried to obtain the final product, PEG-PBAE.

Thz–PPM was prepared using the thin-film dispersion method (26, 27). Firstly, PPM and Thz were suspended in 10 ml acetone at a proportion of 10:3. Then, acetone was removed using reduced pressure at 42°C to acquire a homogeneous film, and it was dried in a vacuum oven for 12 h to remove residual acetone. Secondly, the film layer was dispersed and fully hydrated using deionized water and ultrasound. Finally, the insoluble drug was removed with a desktop centrifuge (DM0412E; Beijing Baiyang Medical Equipment Co., Ltd., Beijing, China) at 10,000 rpm for 15 min and filtered through a 0.45-μm membrane to obtain the final product, Thz–PPM.

#### 2.2.2 Preparation of Thz–HPPMc

Referring to related literatures (28). The Thz/HA-coated PPM complex (Thz–HPPMc) was prepared by electrostatic interaction. Briefly, the HA solution (1.0 mg/ml) was slowly added dropwise into the Thz–PPM solution while stirring at 800 rpm, with a mass ratio of HA to drug-loaded micelles of 2:1. The mixed solution was stirred for 20 min, washed with deionized water, and the unconnected HA was removed by ultracentrifugation. Finally, Thz–HPPMc was obtained under vacuum. According to the above-described method, blank HPPMc without Thz, C6-loaded HPPMc (C6-HPPMc), and Dir-loaded HPPMc (Dir-HPPMc) were prepared.

### 2.3 Characterization of HPPMc

A differential scanning calorimetry (DSC) experiment was conducted using a differential scanning calorimeter (DSC 214; Netzsch, Bavaria, Germany). Weighted samples were placed into aluminum crucibles and measured from 45°C to 350°C under a nitrogen atmosphere.

### 2.4 Characterization of Thz–HPPMc

The micromorphology of Thz–HPPMc was evaluated using a JEM-2100 transmission electron microscope (TEM) (JEOL Ltd., Tokyo, Japan). The Thz–HPPMc solution was dripped on the special copper net for electron microscopy, and about 200 μl of 2% (*w*/*v*) phosphotungstic acid was added. After drying at room temperature for 5 min, the product was tested with TEM.

The zeta potential and particle size of Thz–HPPMc were examined using a Zetasizer Nano-ZS particle size analyzer (Zetasizer Nano-ZS90; Malvern Instruments, Malvern, UK). The drug loading (DL) capacity and encapsulation efficiency (EE) of Thz–HPPMc were detected using high-performance liquid chromatography (HPLC; Waters 2695, Milford, MA, USA) with UV detection at 301 nm. The DL and EE of Thz–HPPMc were calculated using the following formula:


$$\text{EE}\%=\frac{W_{1}}{W_{2}}\times 100\%$$

$$\text{DL}\%=\frac{W_{1}}{W_{1}+W_{3}}\times 100\%$$

where *W*_1_ is the drug-loaded quality in complex solution, *W*_2_ is the input drug quality, and *W*_3_ is the carrier material quality.

### 2.5 *In Vitro* Drug Release Behavior

The *in vitro* release of Thz from Thz–HPPMc under different pH conditions was determined with the dynamic membrane dialysis method (29, 30). Firstly, 2 ml of the Thz–HPPMc solution with/without HAase was placed into a dialysis bag (*M*_w_ = 3,500) against phosphate-buffered saline (PBS) solution (containing 0.5% SDS) at pH 7.4 or 5.5 at 37°C. At the designated time, the release solution was taken out from the medium and replaced by the same amount of a new PBS solution. The concentration of Thz released from the medium was detected using HPLC. The cumulative release (*C*_r_) was calculated according the formula in the literature (23).

### 2.6 HPLC Analysis

The concentration of Thz was determined with HPLC (Waters2695) using a Discovery C18 Column (Sigma-Aldrich, St. Louis, MO, USA) at 25°C. The mobile phase is methanol/water (65:35, *v*/*v*) containing 150 μl trimethylamine/500 ml water, and the flow rate is 1.0 ml/min. Thz was detected at 301 nm. A linear response was obtained in the concentration range between 0.2 and 100 μg/ml. For the release samples, 200 μl was injected and the actual weight of Thz was determined from the calibration curve.

### 2.7 *In Vitro* Stability of Thz–HPPMc

The stability of the complex in serum was investigated by measuring its particle size. Firstly, Thz–HPPMc was incubated with FBS for 0, 0.5, 1, 2, 4, 8, and 12 h at 37°C. The particle size of HPPMc was determined using the principle of dynamic light scattering (DLS) (Vasco, Micromeritics, Norcross, GA, USA).

The stability of the complex in the presence of SDS as a destabilizing agent was measured (31, 32). Of SDS, 90 μl (50 mg/ml) was added to 3 ml of Thz–HPPMc (0.5 mg/ml). The complex was then monitored using DLS.

### 2.8 Cytotoxicity Study

The cell cytotoxicity of blank polymer micelles or Thz–HPPMc was measured by the MTT assay with the use of MS (33). Firstly, suspension solution of MS (10,000 cells per well) was added into 96-well plates and incubated for 24 h. After incubation, the cells were treated with 10 μl of different concentrations of HPPMc without Thz (ranging from 10 to 500 μg/ml), Thz–HPPMc (Thz concentration ranging from 1 to 50 μM) under different pH values (pH 7.4 or 5.5), and the control separately. The control group was without the preparation. After incubation for 48 h, the MTT reagent was added for another 4 h. Finally, the resultant purple formazan crystals were dissolved in triplex solution (5% isobutanol, 0.01 M HCl, and 10% SDS). The absorbance of every well was measured using an enzyme labeling instrument (Synergy H1; BioTek Instruments, Winooski, VT, USA) at 570 nm. The cell viability rates were calculated according to the following formula:


$$\text{Cell viability}=\frac{A_{\text{sample}}}{A_{\text{control}}}\times 100\%$$

where *A*_sample_ and *A*_control_ are the absorbance values of the experimental and control groups, respectively. The Origin statistical software program (Origin 9.1; OriginLab, Northampton, MA, USA) was used to calculate the IC_50_ values of the different groups.

### 2.9 Study on the Intracellular Uptake of HPPMc

The C6–HPPMc solution (C6–HPPMc was prepared according to the film dispersion method in *Section 2.2.2*) was prepared to evaluate the intracellular uptake with fluorescence microscopy (Leica Microsystems™ DM IL LED, CMS GmbH, Wetzlar, Germany). After 12 h of incubation under normal culture conditions, MS were digested into a single-cell suspension with trypsin, and the single-cell suspension was inoculated into the culture plate and cultured at 37°C. Then, the medium was replaced with DMEM containing C6-HPPMc. Subsequently, the sample solution was incubated for 0.5, 2, and 4 h in pH 7.4 and 5.5 serum-free medium. After incubation, cold (4°C) PBS was used to terminate the intracellular uptake. Eventually, the cells were evaluated with the fluorescence microscope. Meanwhile, the mean fluorescence intensity was detected using flow cytometry (Guava EasyCyte™ 8HT; Millipore Corporation, Darmstadt, Germany) (34, 35).

### 2.10 Cellular Uptake Pathway Experiment

MS were digested by trypsin to prepare a single-cell suspension. The cells were inoculated in six-well plates at an initial density of 25,000 cells for 12 h incubation. Then, after adding 5 mg/ml excess HA into the specified well for 30 min and discarding the culture medium, the medium containing C6-PPM or C6-HPPMc was seeded in each plate. The cell uptake was terminated with cold (4°C) PBS. After centrifuging and discarding the supernatant, the cells were resuspended in 4% formaldehyde solution prepared with fresh 500 μl cold PBS. The results were analyzed using flow cytometry (36, 37).

### 2.11 Lysosome Escape of HPPMc

To understand the distribution of Thz–HPPMc in the cells, an MS single-cell suspension was added into six-well plates and incubated overnight. The cells were washed with PBS. Then, the cells were treated with 2 ml medium containing C6-HPPMc at 37°C for 0.5 and 2 h. Thereafter, the lysosomes and nuclei were treated with Lyso-Tracker Red and Hoechst 33258 solutions, respectively. Subsequently, the cells were washed with PBS and fixed in 4% paraformaldehyde. The final phenomenon was observed and photographed with a confocal laser scanning microscope (CLSM; STELLARIS, Leica, Wetzlar, Germany).

### 2.12 *In Vivo* Imaging

The MCF-7 cell suspension (5 × 10^6^ cells/200 μl) was prepared with 0.9% normal saline. Afterwards, each nude mouse was inoculated and the left chest wall skin was cut about 1.0 cm to expose the second pair of breast fat pads, and then 0.2 ml of the cell suspension was injected. When the tumor grew to 100–300 mm^3^, the preparation group was used for treatment.

The fluorescent dye Dir was retained in PPM (Dir-PPM) and HPPMc (Dir-HPPMc) to study the tumor-targeting effects of the different agents. BALB/c nude mice were randomly divided into three groups: control, Dir-PPM, and Dir-HPPMc. The control group was injected with PBS. At the indicated time points after injection, the mice were anesthetized with 5% chloral hydrate and transferred to a small-animal *in vivo* imager to observe the fluorescence distribution. After sacrificing the mice, the liver, heart, spleen, lung, and kidney were harvested and subjected to a small-animal living imager (Smart Imaging System, NEWTON 7.0; Vilber, Collégien, France) to measure the fluorescence intensity of the isolated organs.

### 2.13 *In Vivo* Antitumor Efficiency

About 8 days after inoculation, the size of the tumor in the right axilla of nude mice reached part of the corresponding volume requirements. BALB/c nude mice were randomly divided into six groups (*n* = 6 each). The following six groups of preparations were injected into mice *via* the tail vein (Dox = 4 mg/kg, Thz = 16 mg/kg) (38, 39): 1) normal saline; 2) Dox; 3) free Thz; 4) free Dox+Thz; 5) free Dox+Thz–PPM; and 6) free Dox+Thz–HPPMc. The animals were injected every 4 days for three consecutive times. After administration of the injections, the animals were observed until the 21st day. In addition, the body weight and size of the tumor were measured after each administration. The tumor inhibition rates (TIR%) were calculated as follows:


$$\text{TIR}\%=\frac{W_{c}-W_{t}}{W_{c}}\times 100\%$$

where *W*_c_ and *W*_t_ are the tumor weights of the control group and the tested group, respectively.

### 2.14 Statistical Analysis

All experiments were performed at least three times. The data are presented as the mean (in percent) ± SD. The results are indicated as average plus or minus SD. SPSS version 22.0 statistical software program (Microsoft, Redmond, WA, USA) was used to perform statistical analysis. Analysis of variance (ANOVA) and Student’s *t*-test were used to examine differences between groups. Statistically significance was set as *p < 0.05 and **p < 0.01.

## 3 Results and Discussion

### 3.1 Characterization of HPPMc

**Figure 1:**
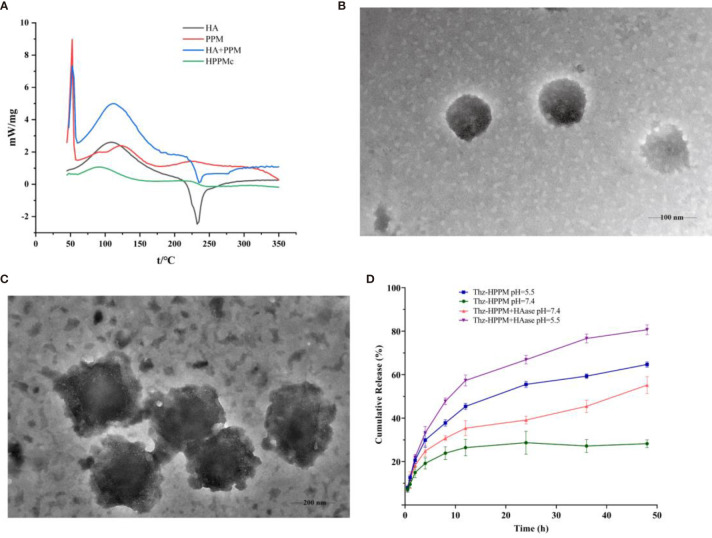
Characterization of HPPMc and Thz–HPPMc. **(A)** Differential scanning calorimetry (DSC) curves of HA, PPM, mixture of HA and PPM, and HPPMc. **(B)** TEM image of Thz–HPPMc (*scale bar*, 100 mm). **(C)** TEM image of Thz–HPPMc (*scale bar*, 200 mm). **(D)** Release profile of Thz from Thz–HPPMc and Thz–HPPMc+HAase at pH values of 7.4 and 5.5. Data shown are the mean ± SD (*n* = 3). *HA*, hyaluronic acid; *PPM*, PEG-poly(β-amino ester) micelle; *HPPMc*, HA-coated PPM complex; *Thz*, thioridazine; *HAase*, hyaluronidase.

DSC is a simple and rapid method used to obtain the information on the interaction between substances (40, 41). **Figure 1A** depicts the DSC curves of HA, PPM, physical mixtures of HA and PPM, and the HA-coated complex. As shown in **Figure 1A**, at 232°C, the single endothermic peak could be found in the DSC curve of HA, which was basically consistent with the exothermic peak reported in the literature (42). The endothermic peak of PPM at 50°C was attributed to the internal PEG (43). The chief typical peaks of both HA and PPM were observed in the mechanically mixed samples. However, when HA was conjugated to PPM, both characteristic peaks of HA and PEG disappeared in the complex curve. The experimental results of DSC illustrated that HA successfully coated the PEG-PBAE micelles and that HPPMc was not a simple physical mixture of HA and PPM (44).

### 3.2 Characterization of Thz–HPPMc

In order to obtain micelles with better stability, the formulation was optimized from the charge ratio of HA to Thz–PPM. The size and potential of the complex were used as indicators for evaluating the stability of the micelles. As shown in **Supplementary Figure S1**, when the charge ratio was 2.6% (the mass ratio of HA to Thz–PPM was 2:1), the particle size was small and uniform and the potential turned negative. These indicated that the system was stable, and it also proved that HA could be coated on the surface of the drug-loaded micelle. The TEM results showed that Thz–HPPMc was a spheroid and uniform in size (**Figure 1B**). On the other hand, Thz–HPPMc was not smooth on the edges due to the outer covering of HA (**Figure 1C**). In addition, the particle size of Thz-PPM was 218.43 ± 2.36 nm and the zeta potential was −36.07 ± 0.62 mV. Compared with the particle size (105.77 ± 4.10 nm) and zeta potential (30.4 ± 3.41 mV) of Thz–PPM in a previously published article (16), in this study, the particle size of Thz–HPPMc increased and the zeta potential also changed from positive to negative, indicating that the HA coating on the PPM surface was successful. The EE% and DL% of Thz–HPPMc were 88.39 ± 2.06 and 11.96 ± 1.23, respectively.

In order to more intuitively observe the cumulative release behavior of Thz from Thz–HPPMc in a blood environment (pH 7.4) and a lysosome environment (pH 5.5) *in vitro*, the cumulative release curves are displayed in **Figure 1D**. Thz–HPPMc showed a relatively stable release behavior in the release medium (pH 7.4), which released Thz of about 24% in 24 h and 27% in 48 h. Compared with Thz–HPPMc at pH 7.4, the release rates of Thz from Thz–HPPMc in a pH 5.5 release medium were 49% in 24 h and 69% in 48 h. A much faster release rate of Thz was observed at pH 5.5, indicating that the complex was sensitive to pH. In addition, we also examined the drug release effect of the complex in the presence of HAase. As can be clearly observed in **Figure 1D**, in the presence of HAase, the drug release effect exhibited the highest drug release under pH 5.5. Therefore, the complex were nanoparticles with pH- and enzyme-responsive. As shown in **Supplementary Figures S2A, B**, Thz–HPPMc was incubated in serum or SDS solution for different times, but the particle size did not change, indicating that it was quite stable in blood circulation.

### 3.3 Cytotoxicity Evaluation

**Figure 2:**
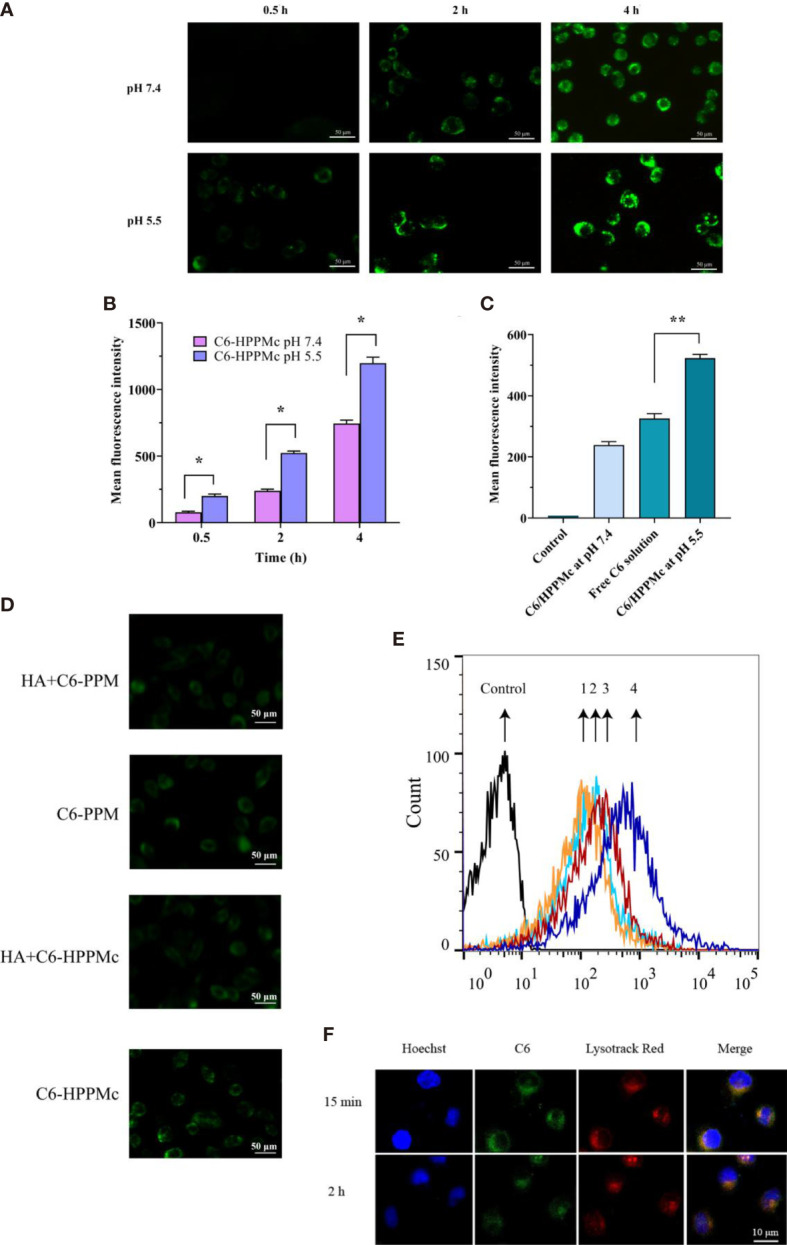
**(A)** Fluorescence microscope images of mammospheres (MS) after treatment with C6-HPPMc for 0.5, 2, and 4 h (at pH 7.4 and 5.5). *Scale bar*, 0.05 mm. **(B)** Results of MS flow cytometry treated with C6-HPPMc at pH 5.5 or 7.4 for 0.5, 2, and 4 h Data shown are the mean ± SD (*n* = 3). **(C)** Fluorescence intensity of MS treated with free C6 and C6-HPPMc for 4 h (pH 7.4 and 5.5). Data shown are mean ± SD (*n* = 3). **(D, E)** Confocal image analysis **(D)** and flow cytometry studies **(E)**. *1*: Incubation with HA for 30 min followed by the application of PPM; *2*: direct addition of PPM; *3*: incubation with HA for 30 min followed by the application of HPPMc; *4*: direct addition of HPPMc. *Scale bar*, 0.05 mm. **(F)** C6-HPPMc endosomal escape and its mechanisms. *Scale bar*, 0.01 mm. *HA*, hyaluronic acid; *PPM*, PEG-poly(β-amino ester) micelle; *HPPMc*, HA-coated PPM complex; *C6-HPPMc*, C6-loaded HPPMc. *p < 0.05, **p < 0.01.

To evaluate the possibility of pH-sensitive complexes to treat BCSCs, we compared the blank HPPMc and Thz–HPPMc at different doses under different pH conditions (pH 5.5 and 7.4) with the MTT assay (29, 45) (**Supplementary Figure S3A**). As shown in **Supplementary Figure S3B**, the cytotoxicity of the Thz and Thz–HPPMc solutions was dose-dependent. The IC_50_ values of Thz–HPPMc at pH 7.4 and 5.5 are described in **Supplementary Table S1**. To investigate the enhanced intracellular uptake of the drug induced by HPPMc, the MS were incubated with C6-HPPMc at different time intervals and pH values. The results of fluorescence microscopy are shown in **Figure 2A**. For both pH values, C6 with a weak fluorescence signal was observed after incubation for 0.5 h. The fluorescent signal increased slightly after 2 h and became stronger after 4 h in the cytoplasm. More importantly, during the same incubation period, the intracellular fluorescence was strong at pH 5.5, but was only weak at pH 7.4. These results suggested that the intracellular uptake behavior of C6 was time-dependent. For further confirmation of the results of fluorescence, we used flow cytometry to analyze the cellular uptake of C6. In **Figure 2B**, at 0.5, 2, and 4 h incubations, the mean fluorescence intensity for HPPMc at pH 7.4 was weaker compared to that with pH 5.5. The results were consistent with those for fluorescence microscopy above. Noticeably (in **Figure 2C**), at pH 5.5 for 2 h, the cell uptake of the C6 solution was significantly different from that of C6-HPPMc, which indicated that the drug could be effectively delivered into the cytoplasm due to pH responsiveness and HA enhancement. The results were also consistent with those of the cytotoxicity test.

Based on all the results, we summarize that, due to the pH response, the PBAE in the HPPMc firstly protonated and the HPPMc then disaggregated, hence releasing more C6 after pre-incubation in pH 5.5 to lead to an extensive cellular uptake of C6-HPPMc by MS. All of the findings demonstrated that HPPMc was effective in delivering drugs into the cytoplasm and improving drug uptake in MS.

To prove the role of the HA receptor in the process of C6-HPPMc uptake by MS cells, MS cells were pre-incubated with excessive free HA for 30 min at 37°C, and C6-PPM or C6-HPPMc was then added for further incubation. As shown in **Figure 2D**, in comparison with C6-PPM, MS treated with C6-HPPMc showed stronger fluorescence, which confirmed that the HA-modified C6-PPM could enhance the cellular uptake of the drug, and the uptake of HPPMc was significantly inhibited by excessive free HA, indicating that free HA competed with HPPMc for the same CD44^+^ cell membrane receptor. However, when MS were incubated with PPM, the uptake did not change significantly with or without CD44 blocking (**Figure 2E**). The results showed that HPPMc could not only selectively target CD44^+^ cells but also internalize into CD44^+^ cells through receptor-mediated endocytosis.

HPPMc was taken up into the cells by the CD44 pathway. After reaching the lysosome, the PBAE in the complex was protonated, causing the structure of the complex to be destroyed and the lysosomal membrane to rupture. Finally, C6 was released into the cytoplasm. As shown in **Figure 2F**, at 15 min, there was strong yellow fluorescence in which C6 and lysozymes were combined, and the yellow fluorescence was significantly attenuated at 2 h. These results further supported that the pH sensitivity of the complex was not destroyed after HA coating. Upon uptake by the CD44 receptor pathway, the complex was transportable *via* the lysosomal pathway. The acidic environment of the lysosome can trigger the release of the drug from the complex.

### 3.4 Imaging *In Vivo*

**Figure 3:**
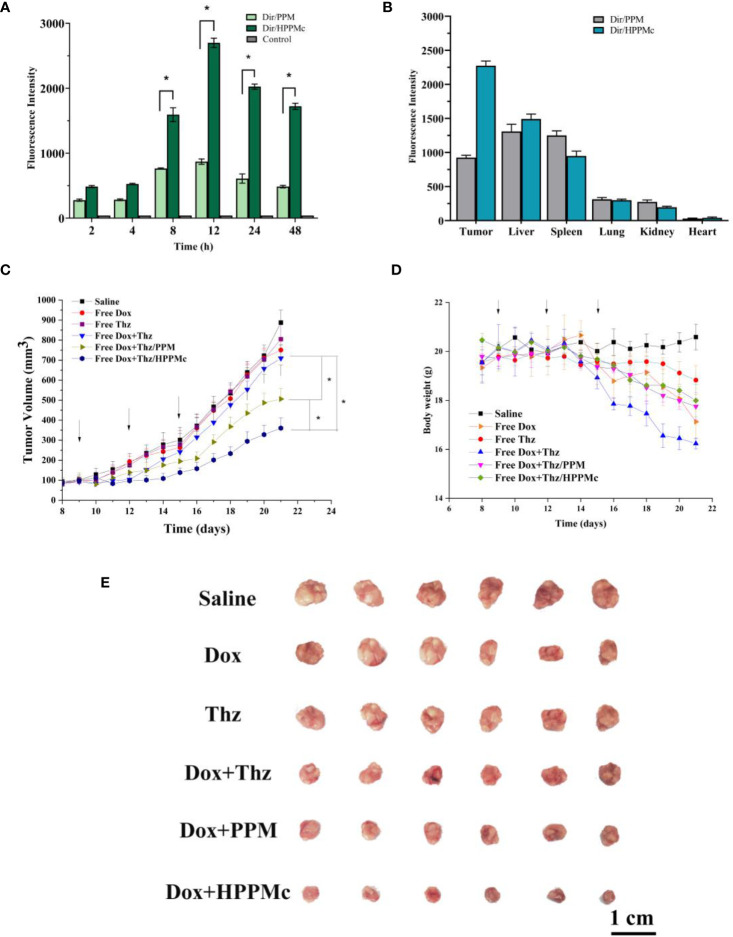
**(A)** Fluorescence intensity of tumor-bearing nude mice at specified times after injection of Dir-PPM, Dir-HPPM, and control. **(B)** Fluorescence intensity of excised organs and tumors 24 h post-injection of the formulation. **(C)** Tumor volume growth curves. Data shown are the mean ± SD (*n* = 6). **(D)** Variations in the body weights of MCF-7 tumor-bearing nude mice treated with different formulations. Data shown are the mean ± SD (*n* = 6). **(E)** Photographs of tumors treated with different the preparation groups. *Dir-PPM*, dimethylindole red-loaded PEG-poly(β-amino ester) micelles; *Dir-HPPM*, Dir-loaded hyalurunic acid-coated PPM complex. *p < 0.05.

To explore the active targeting effect of HPPMc on breast tumors with high CD44 receptor expression, PPM were used as a control to assess the *in vivo* distribution and tumor targeting of Dir-PPM and Dir-HPPMc. Firstly, as expected in **Figure 3A**, both Dir-PPM and Dir-HPPMc could be reached and accumulated in the tumor site within 2–4 h. After 8–12 h, the fluorescence intensities of Dir-PPM and Dir-HPPMc reached the maximum at the tumor site. However, after 24 h, the fluorescence intensities decreased. Compared with PPM, the accumulation of HPPMc was significantly higher at the tumor site. After HA coating, HPPMc had the ability to actively target breast cells with high CD44 expression and increase its accumulation in tumor sites. Furthermore, in **Figure 3B**, Dir-PPM and Dir-HPPMc were mainly distributed in the tumor, liver, lung, and the spleen.

### 3.5 *In Vivo* Antitumor Efficiency

CSCs are the root cause of the drug resistance, recurrence, and metastasis of existing chemotherapy methods. Therefore, tumor-targeted therapies should selectively kill CSCs and tumor cells. To evaluate the antitumor efficiency of Thz–HPPMc, doxorubicin (Dox) as a model drug was added to inhibit tumor cells. Because CSCs are resistant to Dox, the therapeutic effect of Dox on CSCs is greatly reduced (46). Therefore, Thz was combined with Dox to evaluate the anticancer effect. We observed a change trend in the tumor volume and weight of tumor-bearing mice after drug administration. In **Figures 3C, E**, compared with those administered normal saline, mice in the Dox and Thz solution group showed no significant changes, suggesting that Dox and Thz had a weak inhibitory effect on tumor cells. Compared with free Dox+Thz and normal saline, after combination with Dox, the tumor growth rate of BALB/c nude mice decreased after Thz–PPM and Thz–HPPMc administration, showing that micelles and complexes have inhibitory effects on the tumor growth and were clearly higher than those of the free Dox+Thz group. However, Dox+Thz–PPM and Dox+Thz–HPPMc showed different inhibitory effects on tumor in mice, and compared with Dox+Thz–PPM, Dox+Thz–HPPMc showed a significantly enhanced tumor-inhibitory activity. The results showed that micelles significantly inhibited the tumor growth. As shown in **Figure 3D**, different formulations caused significant weight loss in tumor-bearing mice since the start of administration. Because Dox has certain cardiotoxicity, Thz had other side effects in addition to cardiotoxicity. Therefore, both the free Dox and the free Thz caused weight changes in tumor-bearing mice. However, compared with the free Dox and Thz groups, Thz–PPM and Thz–HPPMc combined with Dox resulted in an insignificant weight loss in mice, suggesting that the drug delivery system reduced the toxic and side effects of Thz, thus increasing its tolerance.

Tumors treated with different preparation groups are shown in **Figure 3E**. At the same time, the inhibitory effect of each group of preparations was evaluated with the inhibition rate. The TIRs of free drug Dox+Thz, micelles, and complexes are shown in **Supplementary Table S2**.

## 4 Conclusion

In this study, HPPMc was successfully prepared by electrostatic interaction and Thz–HPPMc was evaluated. After HA modification, the drug-loaded micelles showed regular appearance, round, not smooth, and with particle size larger than 200 nm, negative potential, decent drug loading, and encapsulation efficiency. Moreover, the HA coating did not affect the pH sensitivity of Thz–PPM, and the drug release from the prepared HPPMc could be stimulated in the presence of an acidic pH and enzymes. The acidic environment and HAase in the lysosome caused the depolymerization of the HPPMc to successfully trigger the release of Thz and kill BCSCs. More importantly, the intracellular uptake behavior of the HA-coated PPM exhibited a time-dependent profile, and the C6-entrapped HPPMc showed a more efficient cellular uptake by receptor-mediated endocytosis. *In vivo* imaging experiments demonstrated that HA-coated micelles can specifically target tumor tissues *in vivo* and that drugs can be stored in tumor tissues for a longer time. In antitumor experiments, compared with the Dox and Thz solutions, the Thz–PPM and Thz–HPPMc groups combined with Dox showed an increased tumor inhibition effect of Thz *in vivo* and a decreased toxicity of Thz at the same time. In conclusion, Thz–HPPMc was proven to be a pH-responsive, enzyme-responsive, biocompatible, and CSCs-targeted nanocarrier that specifically recognized and selectively eliminated BCSCs overexpressing CD44.

## Data Availability Statement

The original contributions presented in the study are included in the article/**Supplementary Material**. Further inquiries can be directed to the corresponding author.

## Ethics Statement

The animal study was reviewed and approved by Heilongjiang University of Chinese Medicine.

## Author Contributions

WL, XZ and YN curated the data and prepared the original draft. WL conceptualized the study and helped with software. LJ reviewed and edited the manuscript. JS contributed to visualization and Investigation. LZ helped with software and validation. YW supervised the study. All authors contributed to the article and approved the submitted version.

## Funding

Funds were received from Science Foundation Project of Heilongjiang Province of China (No. LH2021H098), National Natural Science Foundation of China (No. 82074025) and Postgraduate Funds for Heilongjiang University of Chinese Medicine (2020yjscx054).

## Conflict of Interest

The authors declare that the research was conducted in the absence of any commercial or financial relationships that could be construed as a potential conflict of interest.

## Publisher’s Note

All claims expressed in this article are solely those of the authors and do not necessarily represent those of their affiliated organizations, or those of the publisher, the editors and the reviewers. Any product that may be evaluated in this article, or claim that may be made by its manufacturer, is not guaranteed or endorsed by the publisher.
