# Supplementary material for: Hyaluronidase and pH Dual-Responsive Nanoparticles for Targeted Breast Cancer Stem Cells
Source: Front Oncol. 2021 Dec 24;11:760423. doi: 10.3389/fonc.2021.760423 (PMC8739758; doi:10.3389/fonc.2021.760423)
Supplement: Supplementary file 1 [file DataSheet_1.docx]

**Supplementary material**

**Hyaluronidase and pH dual-responsive nanoparticles for targeted breast cancer stem cells**

**Weinan Li ^1†^, Xiaoyu Zhang ^1†^, Yang Nan ^1†^, Li Jia^2^, Jialin Sun^3^, Lina Zhang^1^ and Yanhong Wang^1*^**

Weinan Li, Xiaoyu Zhang, and Yang Nan contributed equally.

^1^School of Pharmacy, Heilongjiang University of Chinese Medicine, Harbin 150040, People's Republic of China

^2^Department of pharmacy, Heze Medical College, Heze 274000, People's Republic of China

^3^Biological Science and Technology Department, Heilongjiang Vocational College for Nationalities, Harbin 150066, People's Republic of China

*Corresponding author: Prof. Y.H. Wang

E-mail: [wang.yanhong@163.com](mailto:wang.yanhong@163.com)

S1 Supplementary results

S1.1 Characterization of Thz-HPPMc

$$\text{Charge ratio}\left( \text{\%} \right)\text{=}\frac{\text{M}_{\text{1}}\text{×0.13}}{\text{M}_{\text{2}}\text{×2.5}}$$

Where, M_1_ represented the quality of PEG-PBAE, 0.13 represents 1 μg PEG-PBAE containing 0.13 nmol amino; M_2_ represented the mass of HA, and 1 μg HA contained the carboxyl group of 2.5 nmol.


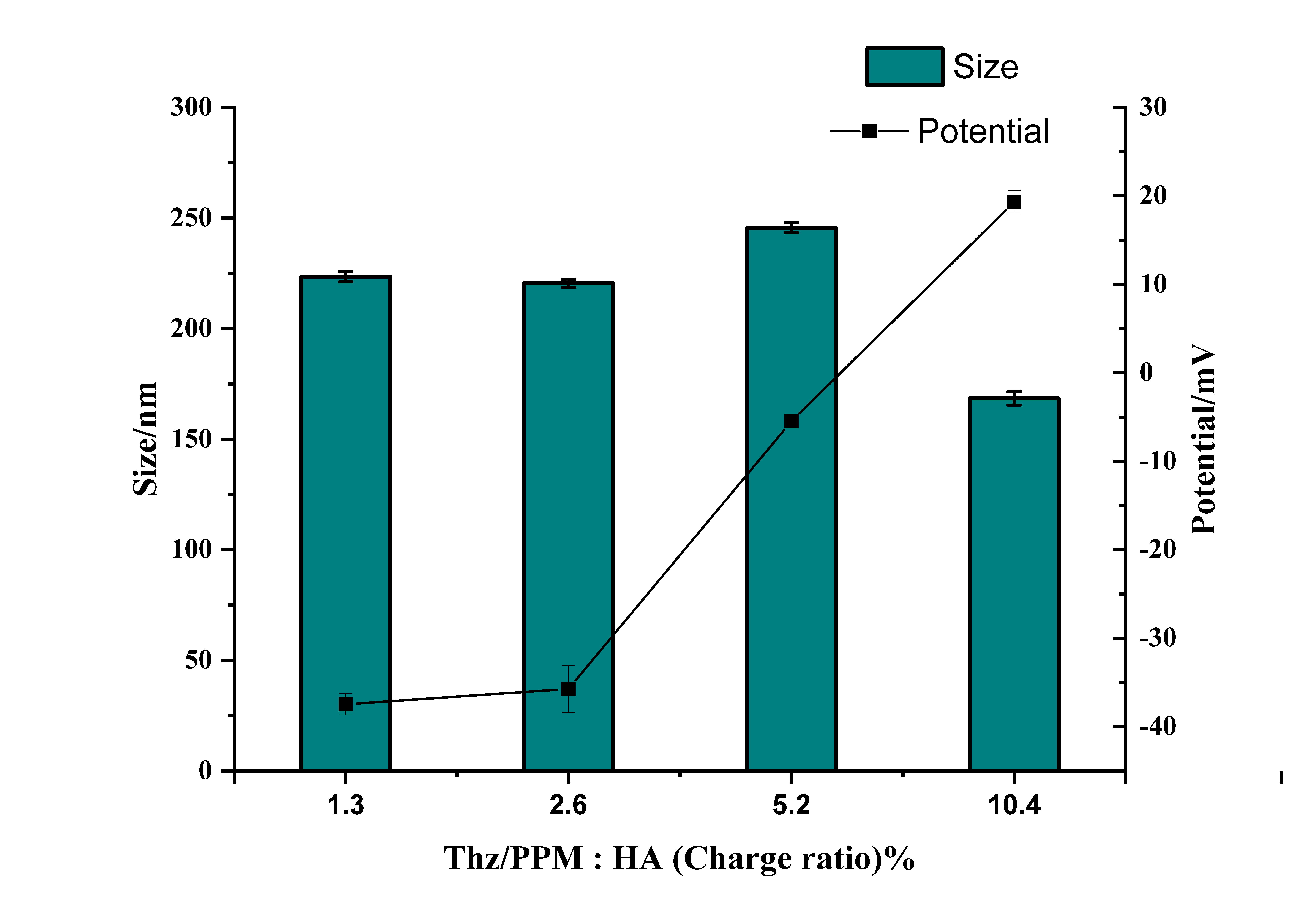


**Fig. S1**. Particle size and potential of the Complex. The charge ratios of HA to Thz/PPM were 1:2, 1:1, 2:1, 4:1, and the charge ratios which were calculated by the formula were 10.4%, 5.2%, 2.6%, 1.3%, respectively.

S1.2 In vitro stability of Thz-HPPMc

In the Fig. S2A, after co-incubation in serum for different times, the particle size of the copolymer did not change significantly. The result showed that HA was attracted to the surface of PPM by static electricity, and various substances in the serum could not destroy the structure of HPPMc. The complex could maintain stability for at least 12 h in serum. In the Fig. S2B, in the presence of sodium dodecyl sulfate (SDS), the particle size of Thz-HPPMc was measured by DLS. The particle size of Thz-HPPMc had no change in SDS solution for 48 h, which indicated that it was quite stable in the blood circulation for at least 48 h.

**B**

**A**


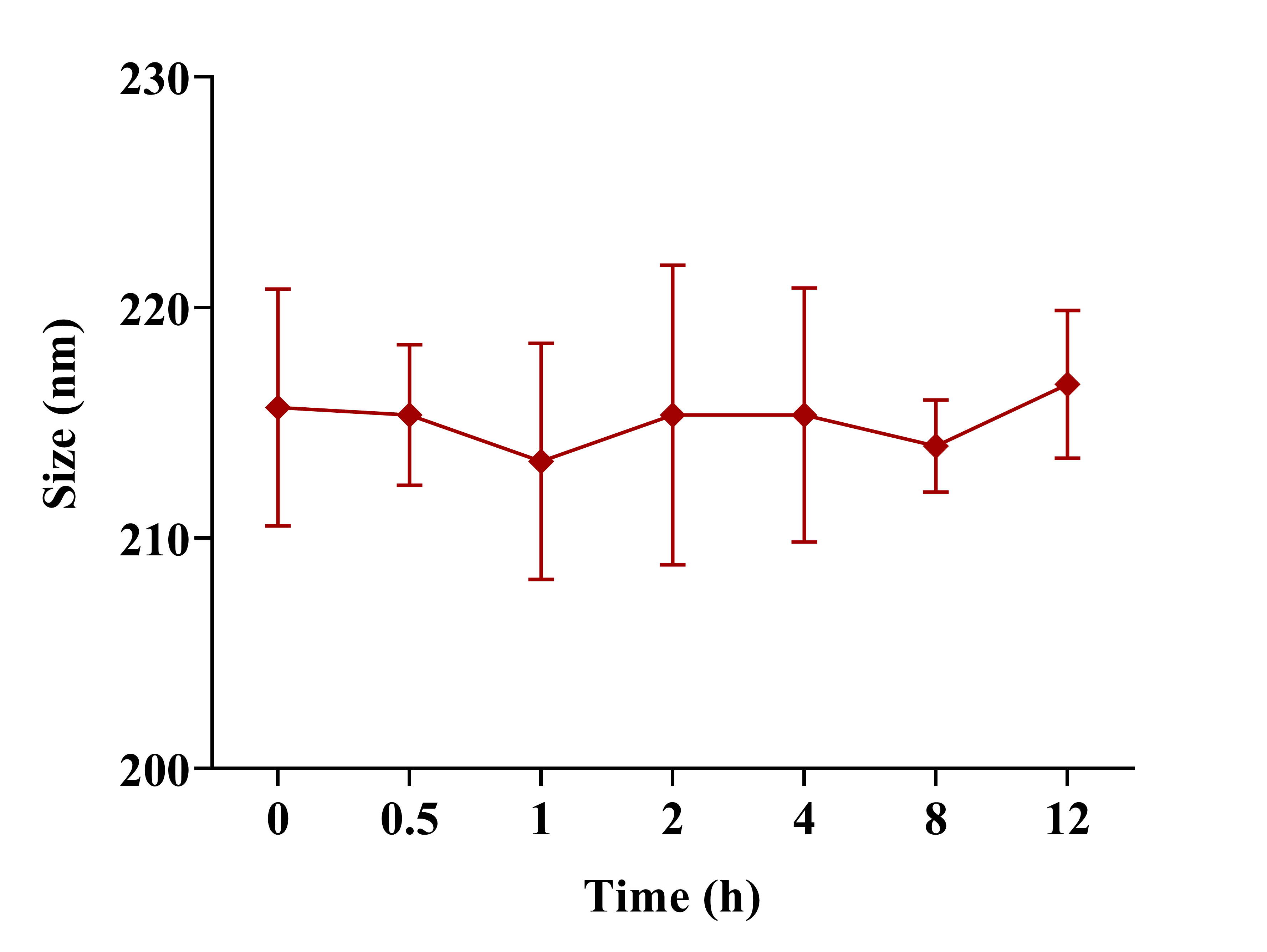

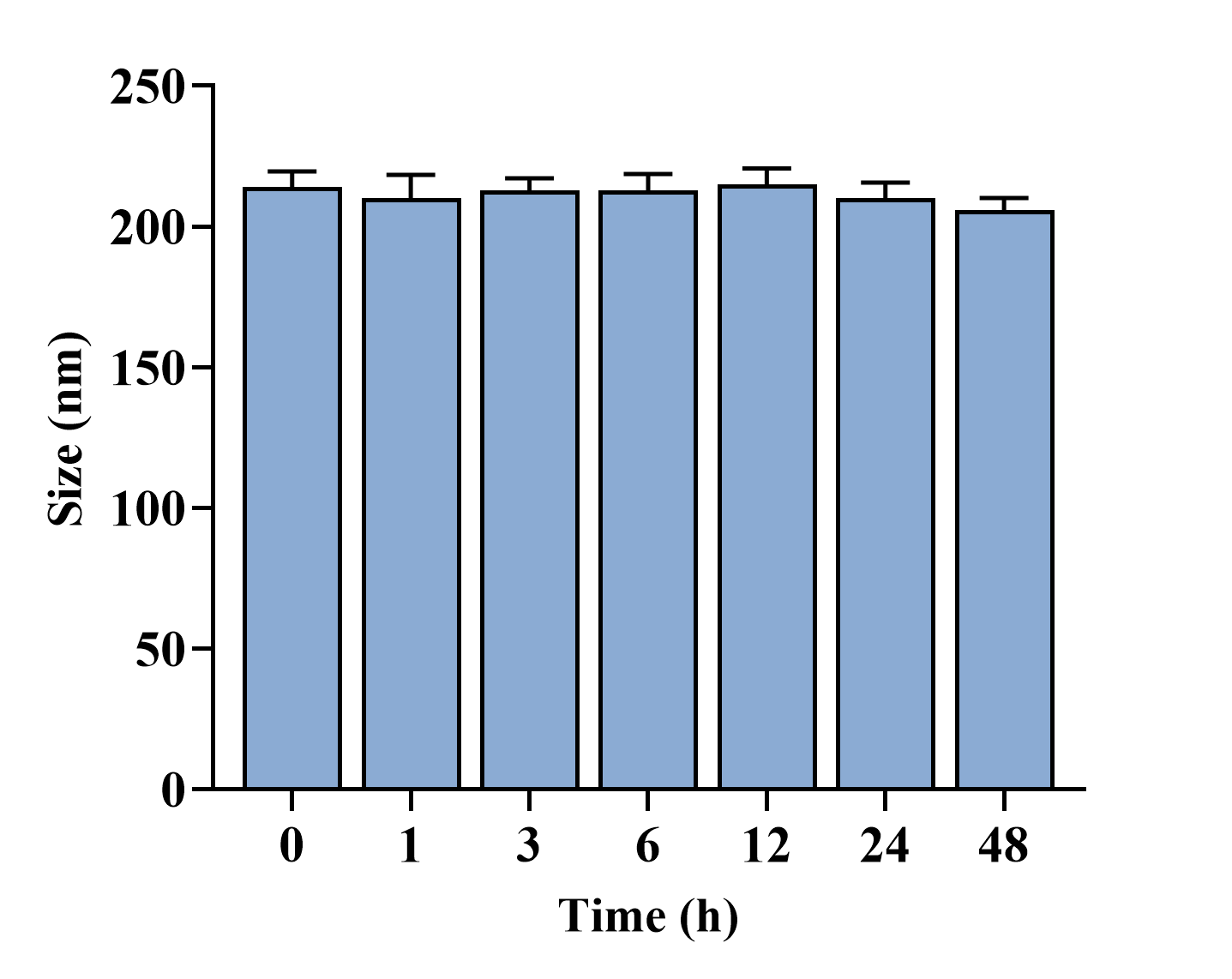


**Fig. S2.** A. Stability of Thz-HPPMc in fetal bovine serum, data shown as mean ± SD, (n = 3). B. Stability of Thz-HPPMc in SDS solution, data shown as mean ± SD, (n = 3).

S1.3 Cytotoxicity study

Anti-CD44-FITC and anti-CD24-PE have been used to identify the surface antigens of MS cells incubated in serum-free medium^[1]^. To evaluate the possibility of pH-sensitive complexes to treat BCSCs, we compared the blank HPPMc and Thz-HPPMc at different doses under different pH conditions (pH 5.5 and 7.4) by MTT assay ^[2,3]^. As described in Fig. 3A, the blank complex showed almost no toxicity at up to 500 μg/mL of complex concentration for the MS at different pH values, indicating that the blank HPPMc was biocompatible with MS for further cytotoxicity study of drug-loaded complex. As in Fig. 3B, the cytotoxicity of Thz and Thz-HPPMc solution performed dose-dependent. The IC_50_ values of Thz-HPPMc at pH 7.4 and pH 5.5, are shown in Table S1.

**B**

**A**


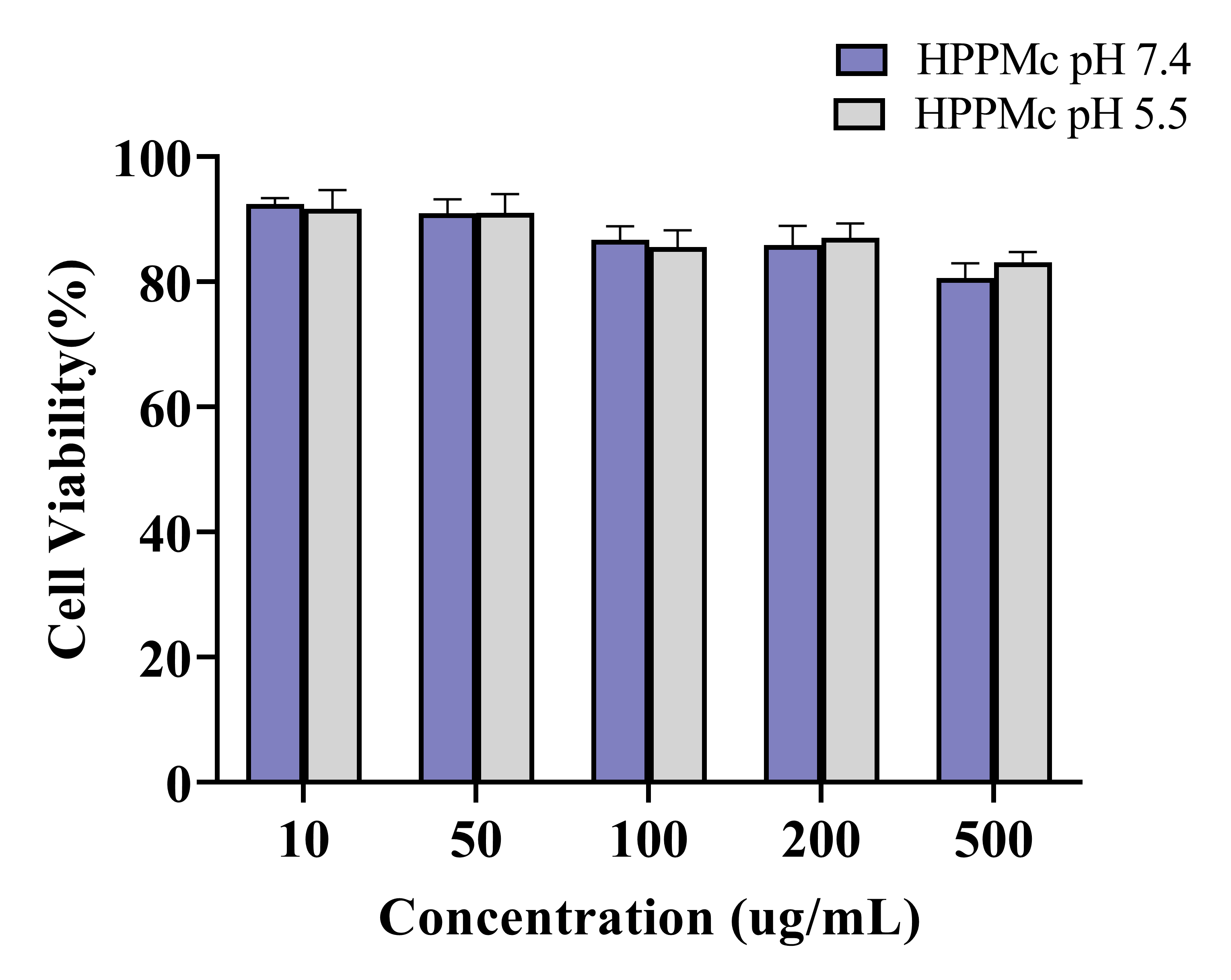

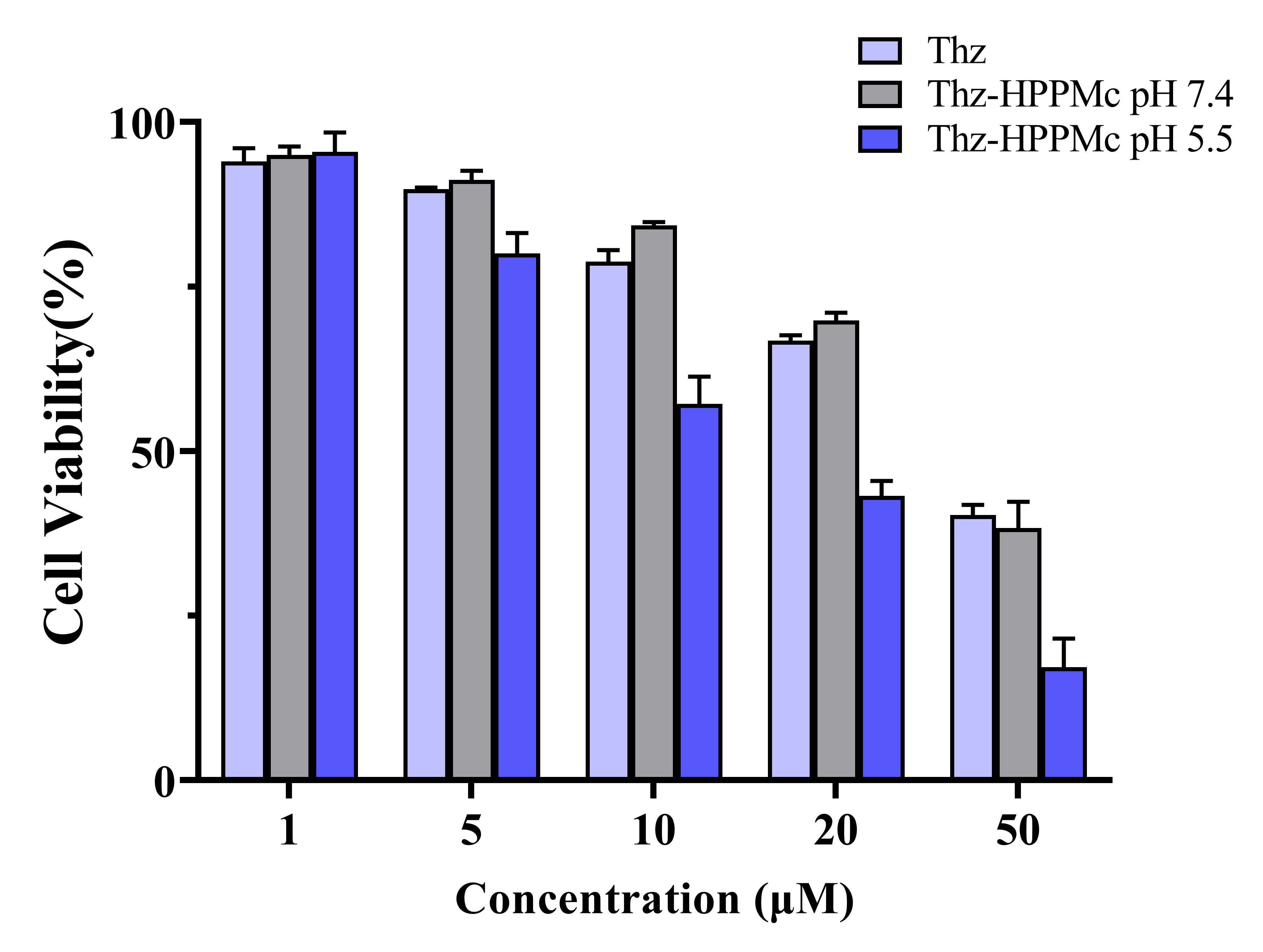


**Fig. S3** Cell viabilities of MS after treatment with (A) blank HPMMc, data shown as mean ± SD, (n = 3). and (B) Thz-HPPMc for 48 h at pH 7.4 and 5.5, data shown as mean ± SD, (n = 3).

This lower cytotoxicity for the Thz-HPPMc at pH 7.4 was apparently attributed to the incomplete release of Thz in the complex, which was consistent with results in vitro release behavior. Meanwhile, it also demonstrated that incubation in pH 5.5 caused the disaggregation of the HPPMc, thereby releasing more Thz and resulting in higher cytotoxicity than that in pH 7.4^[2]^.

**Table S1.** The IC_50_ values of Thz-HPPMc at pH 7.4 and pH 5.5.

|  | Thz | Thz-HPPMc pH 7.4 | Thz-HPPMc pH 5.5 |
| --- | --- | --- | --- |
| IC_50_ (μM) | 21.43 ± 3.74 | 41.09 ± 0.78** | 16.86 ± 5.76* |

**p < 0.01 vs Thz/HPPMc pH 5.5 and Thz solution; *p < 0.05 vs Thz solution

S1.4 In vivo antitumor efficiency

**Table S2.** The TIR of free drug Dox+Thz group, micelles and complexes.

|  | Dox+Thz | PPM | HPPMc |
| --- | --- | --- | --- |
| TIR | 48.65% | 57.35% | 75.91% |

Supplementary references

1. Li W, Sun J, Zhang X, Jia L, Qiao M, Zhao X, et al. Synthesis and Characterization of pH-Responsive PEG-Poly(β-Amino Ester) Block Copolymer Micelles as Drug Carriers to Eliminate Cancer Stem Cells. Pharmaceutics (2020) 12:111. doi:10.3390/pharmaceutics12020111.

2. Sun Y, Li Y, Nan S, Zhang L, Huang H, Wang J. Synthesis and characterization of pH-sensitive poly(itaconic acid)-poly(ethylene glycol)-folate-poly(l-histidine) micelles for enhancing tumor therapy and tunable drug release. *J. Colloid Interface. Sci* (2015) 458:119-29. doi: 10.1016/j.jcis.2015.07.008.

3. Ke XY, Lin Ng VW, Gao SJ, Tong YW, Hedrick JL, Yang YY. Co-deliveryof thioridazine and doxorubicin using polymeric micelles for targeting both cancer cells and cancer stem cells. Biomaterials (2014) 35:1096-108. doi: 10.1016/j.biomaterials.2013.10.049.
